# Supplementary material for: mRNA-based VP8* nanoparticle vaccines against rotavirus are highly immunogenic in rodents
Source: NPJ Vaccines. 2023 Dec 22;8:190. doi: 10.1038/s41541-023-00790-z (PMC10739717; doi:10.1038/s41541-023-00790-z)
Supplement: Supplementary file 1 — Supplementary information [file 41541_2023_790_MOESM1_ESM.pdf]

SUPPLEMENTARY INFORMATION

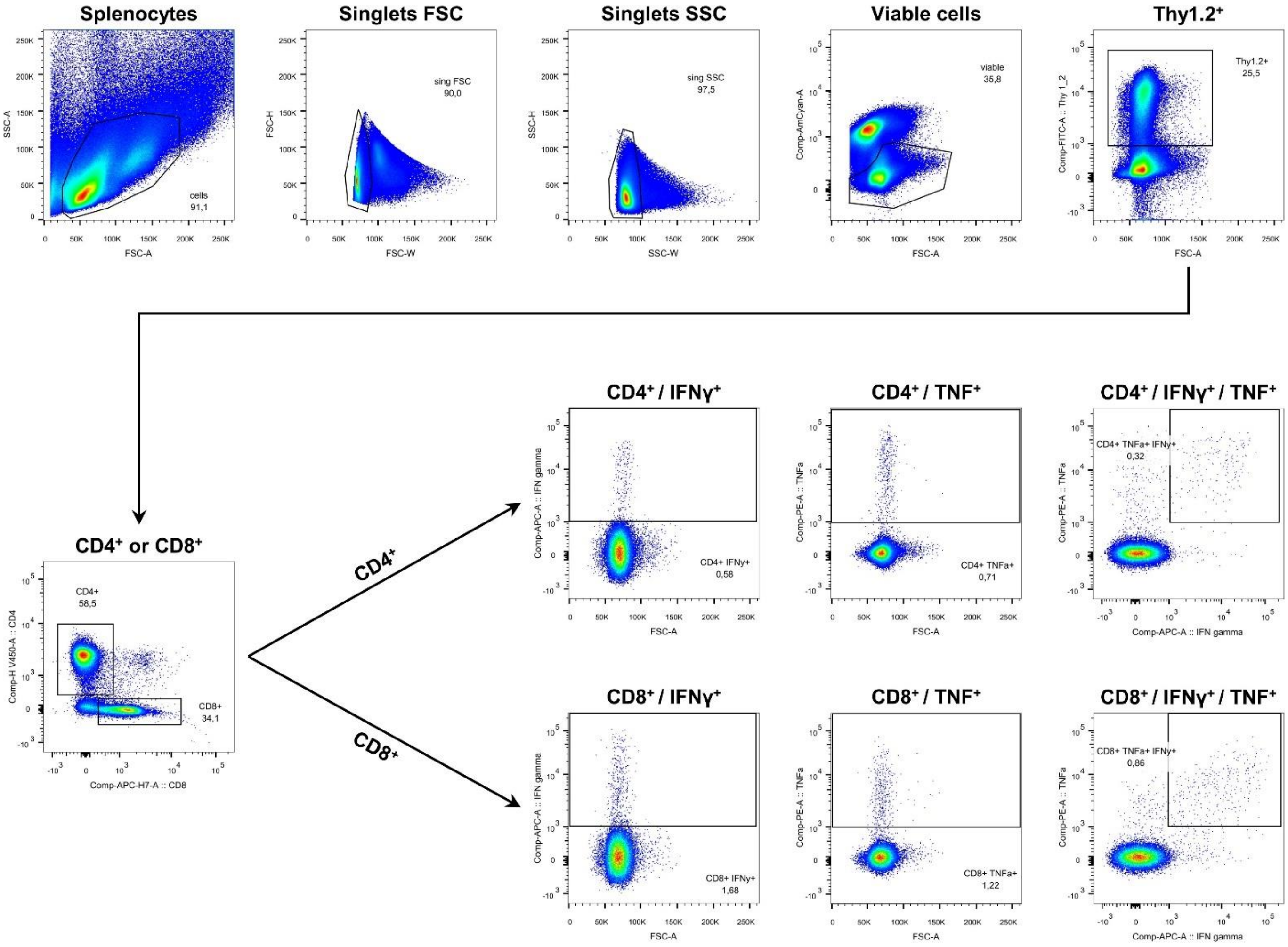

Supplementary Fig. 1 | Gating strategy for T cell analysis. Female BALB/c mice were vaccinated and splenocytes were isolated as described in Fig. 1. Multifunctional IFN-γ/TNF-positive CD4$^+$ and CD8$^+$ T cells were analyzed in splenocytes stimulated with VP8*-specific peptides followed by intracellular cytokine staining and detection by flow cytometry. The flow blots shown illustrate the gating strategy using splenocytes isolated from a mouse vaccinated with P2-VP8* P[8] mRNA vaccine as an example. T cells were characterized as singlets, viable cells, Thy1.2$^+$, CD4$^+$ or CD8$^+$ and subdivided into multifunctional T cell subsets based on their expression of IFN-γ and TNF.

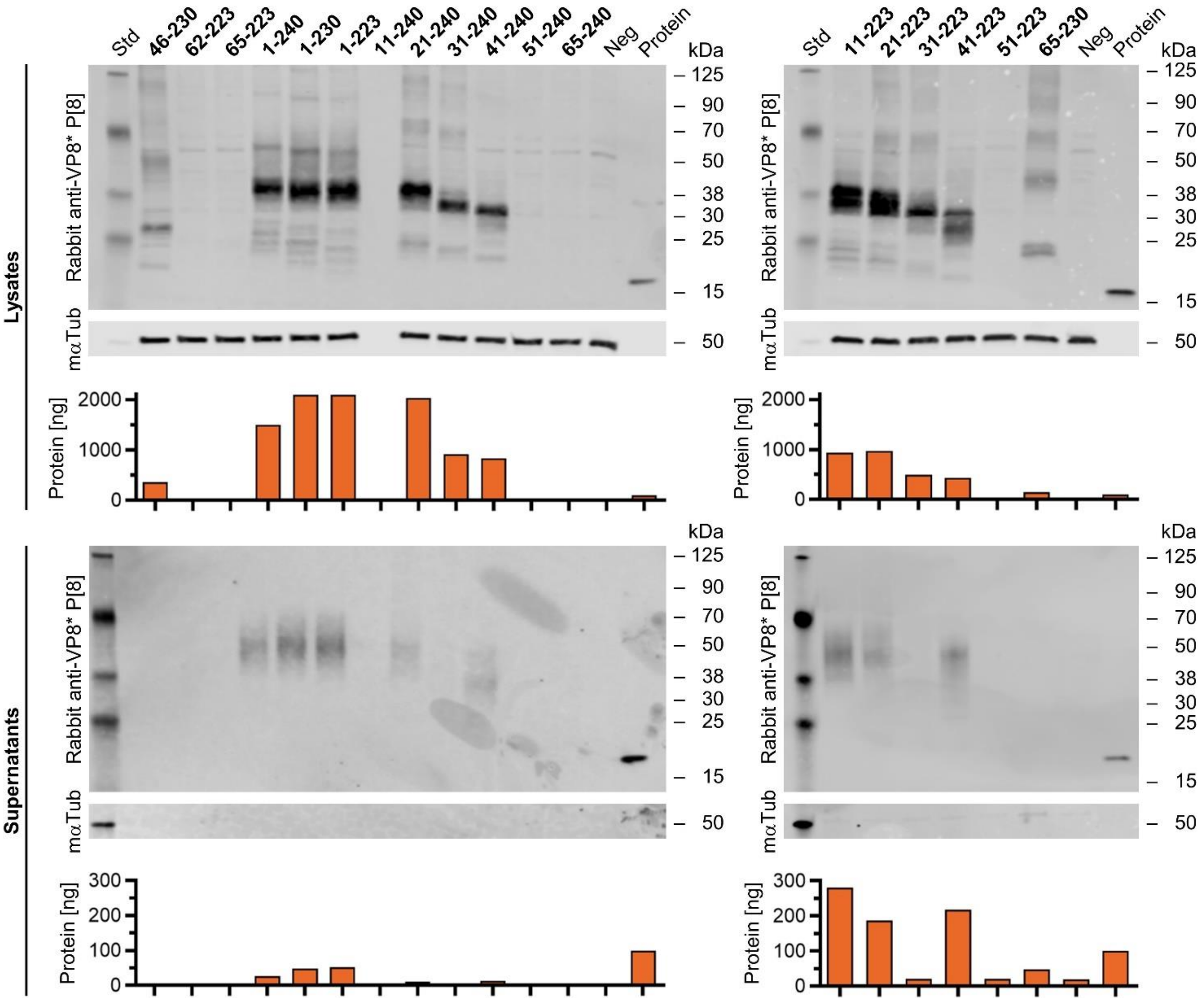

Supplementary Fig. 2 | Expression and secretion levels of proteins translated from pDNAs encoding for different amino acid lengths of VP8* P[8] differed considerably. VP8* expression in HEK 293T cells transfected with different pDNA constructs as indicated was analyzed from cell lysates or supernatants via western blotting 48 hours post transfection. A supernatant or cell lysate from HEK 293T cells transfected with water served as negative control (Neg) and 100 ng of P2-VP8* P[8] protein loaded to the gel was used as positive control (Protein). Tubulin was employed as loading control. Protein expression was quantified using the Image Studio™ Lite version 5.2.5 software and normalized to the signal corresponding to 100 ng of P2-VP8* P[8] protein. mαTub: mouse anti-alpha tubulin antibody.

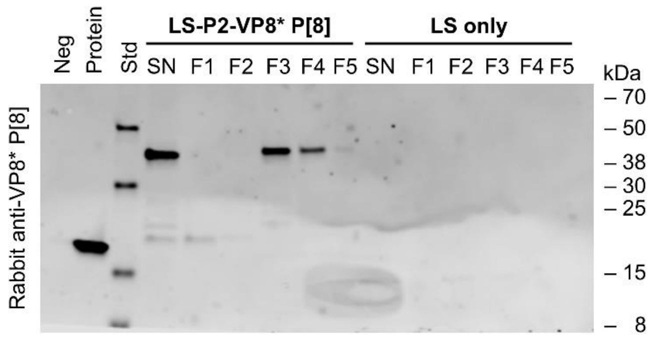

Supplementary Fig. 3 | VP8*-specific signals were only detectable in cellular supernatants transfected with LS-P2-VP8* P[8] mRNA, both before and after ultracentrifugation (compare Fig. 2c). Cell supernatants of HeLa cells transfected with LS-P2-VP8* P[8] or LS only mRNA were subjected to density gradient ultracentrifugation using an isopycnic iodixanol density gradient 48 hours post transfection. After ultracentrifugation, equivalent volumes of five density gradient fractions with increasing iodixanol concentrations (F1 to F5) or unpurified supernatants (SN) were analyzed for VP8* expression via western blotting. A supernatant from HeLa cells transfected with water served as negative control (Neg) and 100 ng of P2-VP8* P[8] protein loaded to the gel was used as positive control (Protein). LS: lumazine synthase.

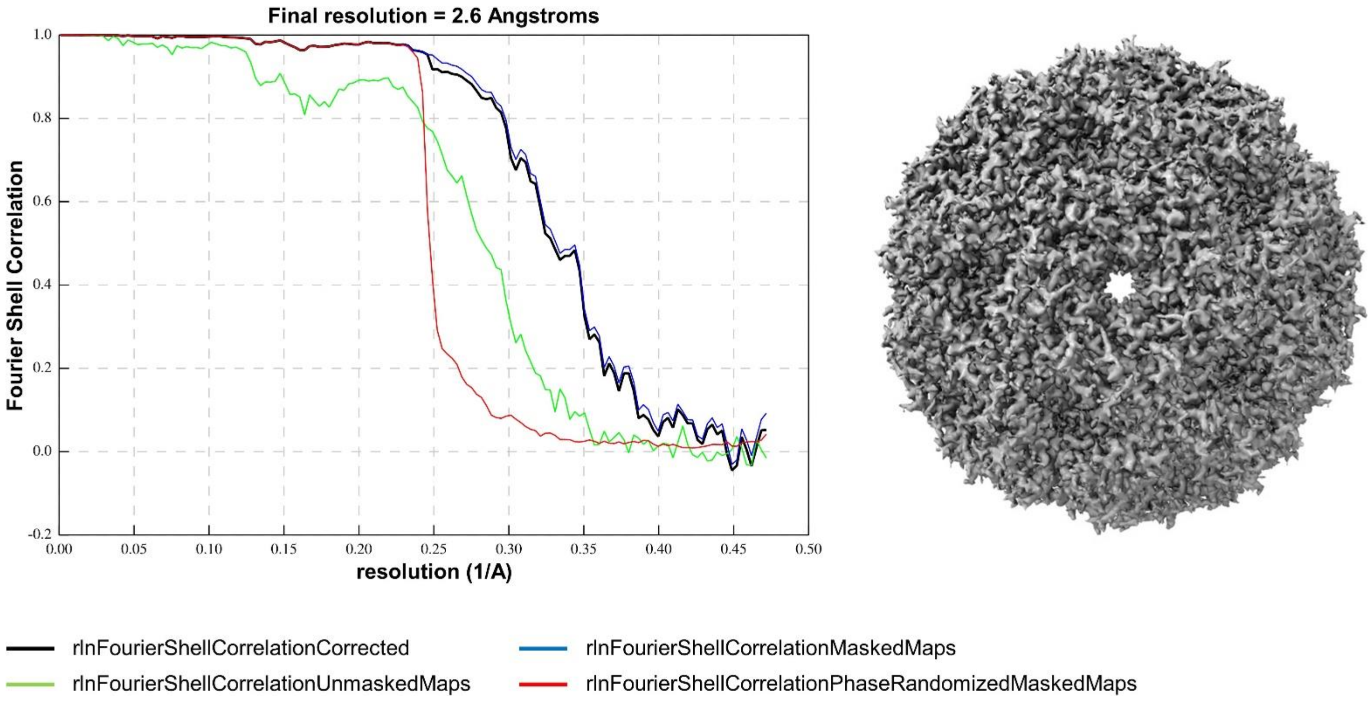

Supplementary Fig. 4 | Resolution estimation for LS-P2-VP8* P[8] cryo-EM structure. Fourier Shell Correlation (FSC) curve for the LS-P2-VP8* P[8] nanoparticle cryo-EM structure (left) along with a 2D snapshot of density map (right).

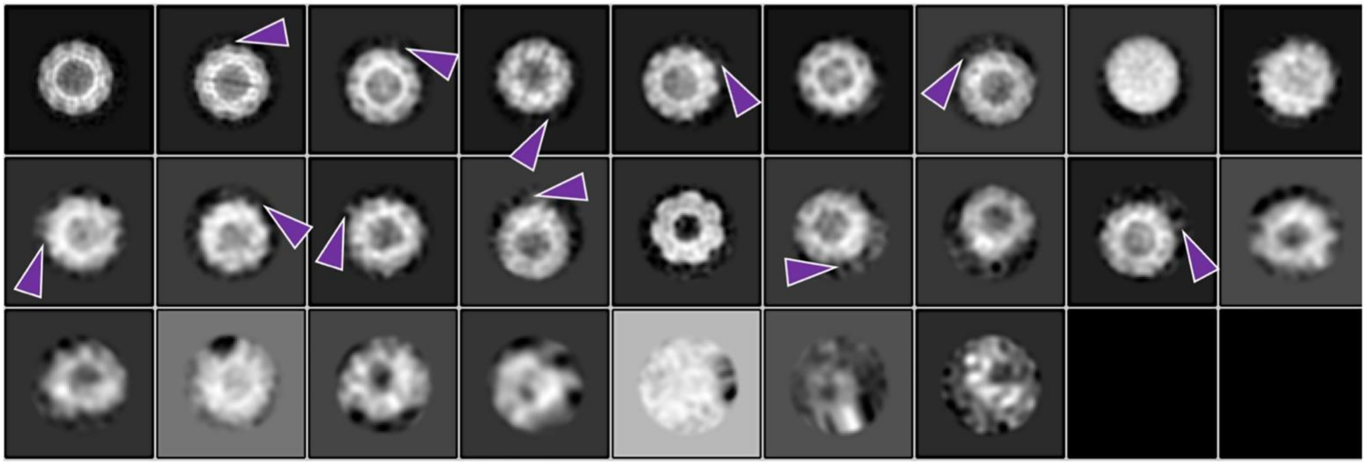

Supplementary Fig. 5 | 2D class averages from LS-P2-VP8* P[8] cryo-EM data demonstrated nonuniform density protruding from nanoparticle surface. Images of averaged classes obtained from unsupervised 2D classification of LS-P2-VP8* P[8] particle images. Purple arrows point to external weak densities seen protruding from the surface of the nanoparticles. White is high density in all the panels.

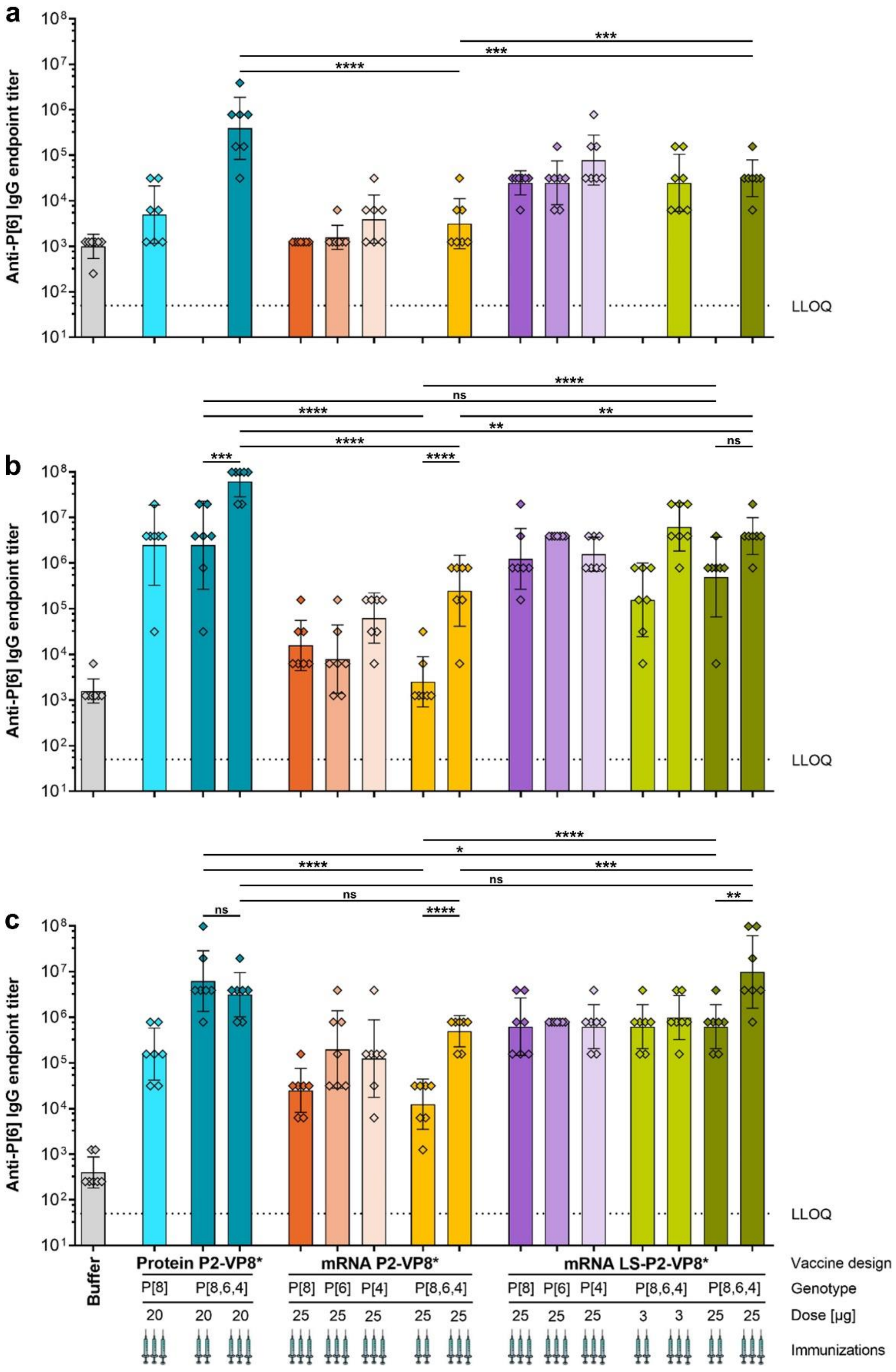

Supplementary Fig. 6 | Trivalent LS-P2-VP8* mRNA vaccine encoding genotypes P[8], P[6] and P[4] of VP8* elicited high titers of binding antibodies against the P2-VP8* P[6] protein in guinea pigs. Female Dunkin-Hartley guinea pigs (n = 7/group) were vaccinated IM either three times on day 0,

day 21, and day 42 or twice on day 21 and day 42 with different doses of monovalent and trivalent alum-adjuvanted P2-VP8* protein vaccines or monovalent and trivalent P2-VP8* or LS-P2-VP8* mRNA vaccines as indicated. The genotype(s) of the encoded VP8* employed for each group, the doses used as well as the number of immunizations administered (indicated by the number of syringe symbols) are displayed beneath each group. Trivalent vaccines are labeled P[8,6,4]. Buffer (0.9% NaCl) vaccinated animals served as negative controls. P2-VP8* P[6]-specific binding antibodies are displayed as endpoint titers for total IgG in serum collected on day 21 **a**, day 42 **b**, or day 70 **c**. Each diamond symbol represents an individual animal and bars depict geometric means with geometric SD. Dotted lines indicate the lower limit of quantification (LLOQ). Individual time points were statistically analyzed using an ordinary one-way ANOVA followed by Šídák's multiple comparison post test. Significant differences between groups are marked by asterisks (*$P<0.05$, ** $P<0.01$, *** $P<0.001$, **** $P<0.0001$; ns: not significant).

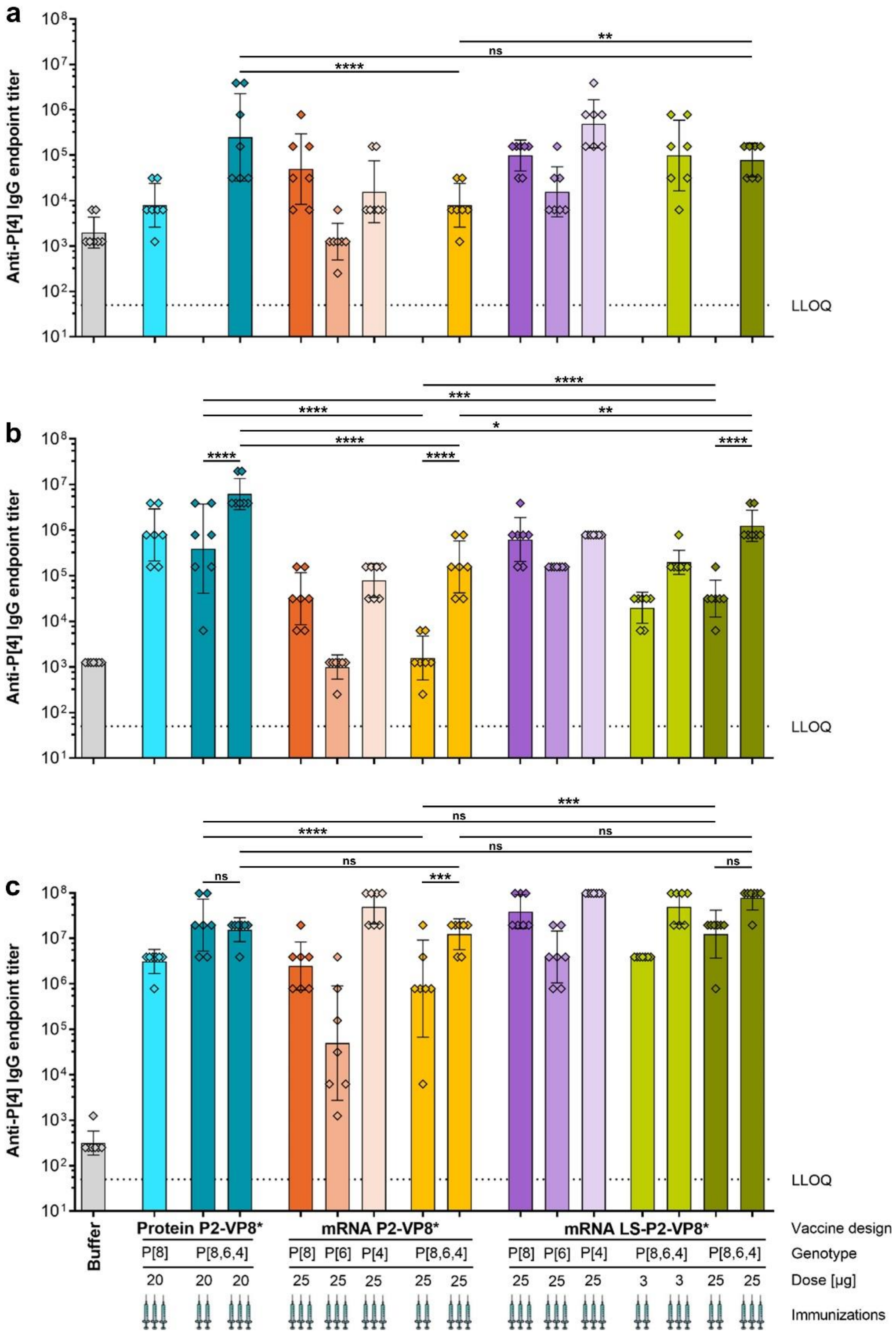

Supplementary Fig. 7 | Trivalent LS-P2-VP8* mRNA vaccine encoding genotypes P[8], P[6] and P[4] of VP8* elicited high titers of binding antibodies against the P2-VP8* P[4] protein in guinea pigs. Female Dunkin-Hartley guinea pigs (n = 7/group) were vaccinated IM either three times on day 0,

day 21, and day 42 or twice on day 21 and day 42 with different doses of monovalent and trivalent alum-adjuvanted P2-VP8* protein vaccines or monovalent and trivalent P2-VP8* or LS-P2-VP8* mRNA vaccines as indicated. The genotype(s) of the encoded VP8* employed for each group, the doses used as well as the number of immunizations administered (indicated by the number of syringe symbols) are displayed beneath each group. Trivalent vaccines are labeled P[8,6,4]. Buffer (0.9% NaCl) vaccinated animals served as negative controls. P2-VP8* P[4]-specific binding antibodies are displayed as endpoint titers for total IgG in serum collected on day 21 **a**, day 42 **b**, or day 70 **c**. Each diamond symbol represents an individual animal and bars depict geometric means with geometric SD. Dotted lines indicate the lower limit of quantification (LLOQ). Individual time points were statistically analyzed using an ordinary one-way ANOVA followed by Šídák's multiple comparison post test. Significant differences between groups are marked by asterisks (*$P<0.05$, ** $P<0.01$, *** $P<0.001$, **** $P<0.0001$; ns: not significant).

Supplementary Table 1 | Cryo-EM data collection, refinement and validation statistics.

	LS-P2-VP8* P[8] nanoparticles (EMDB-28807), (PDB 8F25)
**Data collection and processing**	
Magnification	130000×
Voltage (kV)	300 kV
Electron exposure (e⁻/Å^2)	284.8
Defocus range (μm)	-0.75-3
Pixel size (Å)	0.53
Symmetry imposed	Icosahedral
Initial particle images (no.)	135718
Final particle images (no.)	1936
Map resolution (Å)	2.61 (0.143)
FSC threshold	
Map resolution range (Å)	-
**Refinement**	
Initial model used (PDB code)	5MPP
Model resolution (Å)	2.6
FSC threshold	
Model resolution range (Å)	-
Map sharpening *B* factor (Å^2)	-81.9253
R.m.s. deviations	
Bond lengths (Å)	0.007
Bond angles (°)	0.685
Validation	
MolProbity score	4.04
Clashscore	0.0
Poor rotamers (%)	
Ramachandran plot	
Favored (%)	96.04
Allowed (%)	3.96
Disallowed (%)	0

Supplementary Data | Comprehensive information on the statistical analyses. The accompanying Excel file provides a detailed summary of ANOVA results including multiple comparison testing for each statistical analysis performed in this study.

SOURCE DATA FIGURES

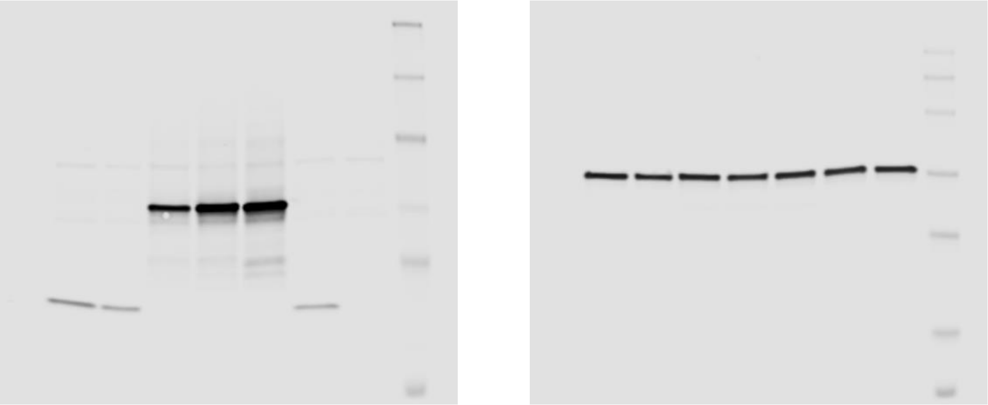

Uncropped western blotting images (left: rabbit anti-VP8* P[8] signal; right: mαTub signal) for Fig. 2b (lysates)

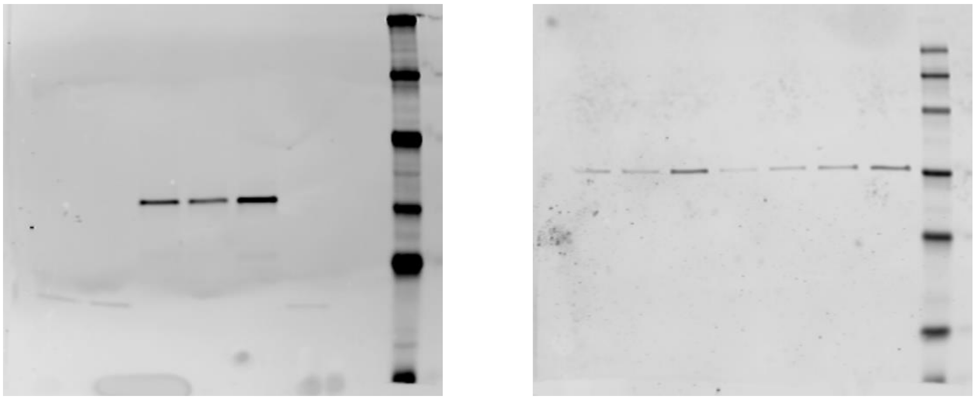

Uncropped western blotting images (left: rabbit anti-VP8* P[8] signal; right: mαTub signal) for Fig. 2b (supernatants)

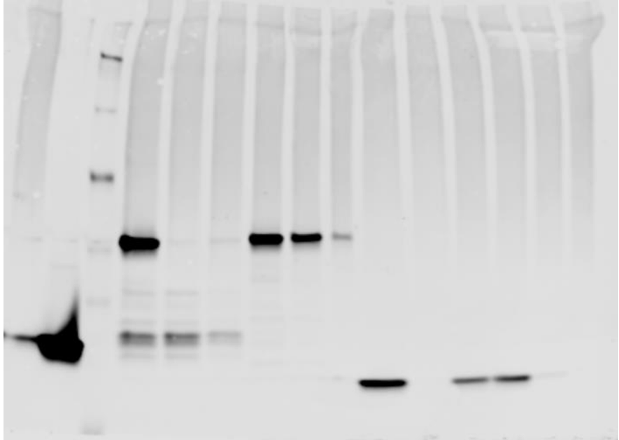

Uncropped western blotting image for Fig. 2c
