# Supplementary material for: mRNA-based VP8* nanoparticle vaccines against rotavirus are highly immunogenic in rodents
Source: NPJ Vaccines. 2023 Dec 22;8:190. doi: 10.1038/s41541-023-00790-z (PMC10739717; doi:10.1038/s41541-023-00790-z)
Supplement: Supplementary file 4 — REPORTING SUMMARY [file 41541_2023_790_MOESM4_ESM.pdf]

nature portfolio

Corresponding author(s): Susanne Rauch

Last updated by author(s): Apr 14, 2023

Reporting Summary

Nature Portfolio wishes to improve the reproducibility of the work that we publish. This form provides structure for consistency and transparency in reporting. For further information on Nature Portfolio policies, see our Editorial Policies and the Editorial Policy Checklist.

Statistics

For all statistical analyses, confirm that the following items are present in the figure legend, table legend, main text, or Methods section.

n/a	Confirmed	
☐	☒	The exact sample size (*n*) for each experimental group/condition, given as a discrete number and unit of measurement
☒	☐	A statement on whether measurements were taken from distinct samples or whether the same sample was measured repeatedly
☐	☒	The statistical test(s) used AND whether they are one- or two-sided<br>*Only common tests should be described solely by name; describe more complex techniques in the Methods section.*
☒	☐	A description of all covariates tested
☐	☒	A description of any assumptions or corrections, such as tests of normality and adjustment for multiple comparisons
☐	☒	A full description of the statistical parameters including central tendency (e.g. means) or other basic estimates (e.g. regression coefficient) AND variation (e.g. standard deviation) or associated estimates of uncertainty (e.g. confidence intervals)
☐	☒	For null hypothesis testing, the test statistic (e.g. *F*, *t*, *r*) with confidence intervals, effect sizes, degrees of freedom and *P* value noted<br>*Give P values as exact values whenever suitable.*
☒	☐	For Bayesian analysis, information on the choice of priors and Markov chain Monte Carlo settings
☒	☐	For hierarchical and complex designs, identification of the appropriate level for tests and full reporting of outcomes
☒	☐	Estimates of effect sizes (e.g. Cohen's *d*, Pearson's *r*), indicating how they were calculated

Our web collection on statistics for biologists contains articles on many of the points above.

Software and code

Policy information about availability of computer code

Data collection	Leginon software
Data analysis	Image Studio Lite version 5.2.5 software; GraphPad Prism version 9.4.1 for Windows software; Relion (version 3.1) software; UCSF Chimera software; Phenix software suite; FlowJo software

For manuscripts utilizing custom algorithms or software that are central to the research but not yet described in published literature, software must be made available to editors and reviewers. We strongly encourage code deposition in a community repository (e.g. GitHub). See the Nature Portfolio guidelines for submitting code & software for further information.

Data

Policy information about availability of data

All manuscripts must include a data availability statement. This statement should provide the following information, where applicable:

- Accession codes, unique identifiers, or web links for publicly available datasets
- A description of any restrictions on data availability
- For clinical datasets or third party data, please ensure that the statement adheres to our policy

The authors declare that all relevant data supporting the findings of this study are available within the paper and its supplementary information files. Additional information and underlying data are available from the corresponding author upon reasonable request. The cryo-EM data and structure of the LS-P2-VP8* P[8]

nanoparticle generated in this study have been submitted to the Electron Microscopy Data Bank (EMDB) under the accession code EMD-28807 and to the Protein Data Bank (PDB) under the accession code 8F25. These are publicly available as of the date of publication.

Human research participants

Policy information about studies involving human research participants and Sex and Gender in Research.

Reporting on sex and gender	No human research participants were involved in the study.
Population characteristics	No human research participants were involved in the study.
Recruitment	No human research participants were involved in the study.
Ethics oversight	No human research participants were involved in the study.

Note that full information on the approval of the study protocol must also be provided in the manuscript.

Field-specific reporting

Please select the one below that is the best fit for your research. If you are not sure, read the appropriate sections before making your selection.

☒ Life sciences ☐ Behavioural & social sciences ☐ Ecological, evolutionary & environmental sciences

For a reference copy of the document with all sections, see nature.com/documents/nr-reporting-summary-flat.pdf

Life sciences study design

All studies must disclose on these points even when the disclosure is negative.

Sample size	The number of animals per group was chosen to be as low as possible while still allowing scientifically valid statements. Sample sizes were based on our experience and knowledge from previous studies with mRNA-based VP8* rotavirus vaccines in mice and guinea pigs. The sample sizes were selected to provide sufficient power to determine differences between the groups.
Data exclusions	No data were excluded.
Replication	Comparisons of the present experimental findings with our previous studies with mRNA-based VP8* rotavirus vaccines in mice and guinea pigs showed high reproducibility. In addition, some groups from the second guinea pig immunogenicity study were treated exactly the same as some groups from the first guinea pig study, demonstrating a high reproducibility of the experimental findings within the present work.
Randomization	Animals were randomly allocated to groups before the start of the studies.
Blinding	Studies were not blinded. All animal work was performed at contract research organizations (CROs) and neutralizing antibody titers were evaluated at the Cincinnati Children's Hospital Medical Center (CCHMC). CROs and CCHMC had no information on specifics of the mRNA vaccines.

Behavioural & social sciences study design

All studies must disclose on these points even when the disclosure is negative.

Study description	*Briefly describe the study type including whether data are quantitative, qualitative, or mixed-methods (e.g. qualitative cross-sectional, quantitative experimental, mixed-methods case study).*
Research sample	*State the research sample (e.g. Harvard university undergraduates, villagers in rural India) and provide relevant demographic information (e.g. age, sex) and indicate whether the sample is representative. Provide a rationale for the study sample chosen. For studies involving existing datasets, please describe the dataset and source.*
Sampling strategy	*Describe the sampling procedure (e.g. random, snowball, stratified, convenience). Describe the statistical methods that were used to predetermine sample size OR if no sample-size calculation was performed, describe how sample sizes were chosen and provide a rationale for why these sample sizes are sufficient. For qualitative data, please indicate whether data saturation was considered, and what criteria were used to decide that no further sampling was needed.*
Data collection	*Provide details about the data collection procedure, including the instruments or devices used to record the data (e.g. pen and paper, computer, eye tracker, video or audio equipment) whether anyone was present besides the participant(s) and the researcher, and whether the researcher was blind to experimental condition and/or the study hypothesis during data collection.*

Timing	*Indicate the start and stop dates of data collection. If there is a gap between collection periods, state the dates for each sample cohort.*
Data exclusions	*If no data were excluded from the analyses, state so OR if data were excluded, provide the exact number of exclusions and the rationale behind them, indicating whether exclusion criteria were pre-established.*
Non-participation	*State how many participants dropped out/declined participation and the reason(s) given OR provide response rate OR state that no participants dropped out/declined participation.*
Randomization	*If participants were not allocated into experimental groups, state so OR describe how participants were allocated to groups, and if allocation was not random, describe how covariates were controlled.*

Ecological, evolutionary & environmental sciences study design

All studies must disclose on these points even when the disclosure is negative.

Study description	*Briefly describe the study. For quantitative data include treatment factors and interactions, design structure (e.g. factorial, nested, hierarchical), nature and number of experimental units and replicates.*
Research sample	*Describe the research sample (e.g. a group of tagged Passer domesticus, all Stenocereus thurberi within Organ Pipe Cactus National Monument), and provide a rationale for the sample choice. When relevant, describe the organism taxa, source, sex, age range and any manipulations. State what population the sample is meant to represent when applicable. For studies involving existing datasets, describe the data and its source.*
Sampling strategy	*Note the sampling procedure. Describe the statistical methods that were used to predetermine sample size OR if no sample-size calculation was performed, describe how sample sizes were chosen and provide a rationale for why these sample sizes are sufficient.*
Data collection	*Describe the data collection procedure, including who recorded the data and how.*
Timing and spatial scale	*Indicate the start and stop dates of data collection, noting the frequency and periodicity of sampling and providing a rationale for these choices. If there is a gap between collection periods, state the dates for each sample cohort. Specify the spatial scale from which the data are taken*
Data exclusions	*If no data were excluded from the analyses, state so OR if data were excluded, describe the exclusions and the rationale behind them, indicating whether exclusion criteria were pre-established.*
Reproducibility	*Describe the measures taken to verify the reproducibility of experimental findings. For each experiment, note whether any attempts to repeat the experiment failed OR state that all attempts to repeat the experiment were successful.*
Randomization	*Describe how samples/organisms/participants were allocated into groups. If allocation was not random, describe how covariates were controlled. If this is not relevant to your study, explain why.*
Blinding	*Describe the extent of blinding used during data acquisition and analysis. If blinding was not possible, describe why OR explain why blinding was not relevant to your study.*

Did the study involve field work? ☐ Yes ☐ No

Field work, collection and transport

Field conditions	*Describe the study conditions for field work, providing relevant parameters (e.g. temperature, rainfall).*
Location	*State the location of the sampling or experiment, providing relevant parameters (e.g. latitude and longitude, elevation, water depth).*
Access & import/export	*Describe the efforts you have made to access habitats and to collect and import/export your samples in a responsible manner and in compliance with local, national and international laws, noting any permits that were obtained (give the name of the issuing authority, the date of issue, and any identifying information).*
Disturbance	*Describe any disturbance caused by the study and how it was minimized.*

Reporting for specific materials, systems and methods

We require information from authors about some types of materials, experimental systems and methods used in many studies. Here, indicate whether each material, system or method listed is relevant to your study. If you are not sure if a list item applies to your research, read the appropriate section before selecting a response.

Materials & experimental systems

n/a	Involved in the study	
☐	☒	Antibodies
☐	☒	Eukaryotic cell lines
☐	☐	Palaeontology and archaeology
☐	☒	Animals and other organisms
☐	☐	Clinical data
☐	☐	Dual use research of concern

Methods

n/a	Involved in the study	
☐	☐	ChIP-seq
☐	☒	Flow cytometry
☐	☐	MRI-based neuroimaging

Antibodies

Antibodies used

For ELISA: biotinylated rat anti-mouse IgG1 antibody (1:300; BD Biosciences, Cat. 550331); biotinylated rat anti-mouse IgG2a antibody (1:300; BD Biosciences, Cat. 550332); horseradish peroxidase (HRP)-conjugated rabbit anti-guinea pig IgG (H+L) antibody (1:500; Invitrogen, Cat. PA1-28597)
For T cell analysis: FITC rat anti-mouse CD90.2 (Thy1.2) (1:200; BioLegend, Cat. 140304); V450 rat anti-mouse CD4 (1:200; BD Biosciences, Cat. 560468); APC-H7 rat anti-mouse CD8a (1:100; BD Biosciences, Cat. 560182); rat anti-mouse CD16/CD32 (1:100; Invitrogen, Cat. 14-0161-85); APC rat anti-mouse IFN-γ (1:100; BD Biosciences, Cat. 554413); PE rat anti-mouse TNF alpha (1:100; Invitrogen, Cat. 12-7321-82)
For rotavirus neutralization assay: rabbit anti-rotavirus IgG antibody; guinea pig anti-rotavirus antiserum; Horseradish peroxidase (HRP) conjugated rabbit anti-guinea pig IgG (Jackson ImmunoResearch, West Grove, PA, USA); a detailed description of the antibodies is provided in the cited reference: Knowlton, D. R., et al. (1991), https://doi.org/10.1016/0166-0934(91)90013-p
For western blotting: rabbit anti-VP8* P[8]/P[4] or P[6] polyclonal antibodies (1:1,000; generated in this study by Aldevron Freiburg GmbH); guinea pig anti-LS-P2-VP8* P[8] polyclonal antiserum (1:500, day 196 serum sample from an animal vaccinated with LS-P2-VP8* P[8] mRNA vaccine; generated in this study by Covance Laboratories Ltd.); mouse anti-alpha tubulin antibody (1:1,000; Abcam, Cat. ab7291); goat anti-rabbit IgG IRDye® 800CW or 680RD (1:10,000; LI-COR, Cat. 926-32211 or Cat. 926-68071); donkey anti-guinea pig IgG IRDye® 800CW (1:10,000; LI-COR, Cat. 925-32411); goat anti-mouse IgG IRDye® 680RD (1:10,000; LI-COR, Cat. 926-68070)

Validation

Antibodies were validated and titrated for specificity prior to use in each assay. Information from manufacturer's instructions and previously published methods were taken into account.

Eukaryotic cell lines

Policy information about cell lines and Sex and Gender in Research

Cell line source(s)

HeLa (DSMZ: ACC 57): cervix cells derived from a female Homo sapiens
HEK 293T (DSMZ: ACC 635): embryonal kidney cells derived from Homo sapiens
MA-104 (ATCC: CRL-2378.1): kidney cells derived from Chlorocebus pygerythrus (previously identified as derived from Cercopithecus aethiops

Authentication

HeLa and HEK 293T cells were commercially purchased from DSMZ and authenticated by DSMZ. MA-104 cells were commercially purchased from ATCC and authenticated by ATCC.

Mycoplasma contamination

All cell lines came mycoplasma free from the manufacturer and were routinely tested for mycoplasma contamination after being expanded and used in the lab.

Commonly misidentified lines
(See ICLAC register)

MA-104 was used as a rotavirus susceptible cell line in the serum rotavirus neutralization assay, a widely used standard assay in the rotavirus vaccine development community.

Palaeontology and Archaeology

Specimen provenance

n/a

Specimen deposition

n/a

Dating methods

n/a

☐ Tick this box to confirm that the raw and calibrated dates are available in the paper or in Supplementary Information.

Ethics oversight

n/a

Note that full information on the approval of the study protocol must also be provided in the manuscript.

Animals and other research organisms

Policy information about studies involving animals; ARRIVE guidelines recommended for reporting animal research, and Sex and Gender in Research

Laboratory animals	Female BALB/cAnNRj mice (6-8 weeks old) were obtained from Janvier Labs, France, and were provided and handled by Preclinics Gesellschaft für präklinische Forschung mbH (Potsdam, Germany).<br>Female Dunkin Hartley guinea pigs (6-7 and 10-11 weeks old for first and second study, respectively) were obtained from Envigo RMS, The Netherlands, and were provided and handled by Covance Laboratories Ltd. (now Labcorp, Huntingdon, UK).<br>Female rabbits were provided and handled by Davids Biotechnologie GmbH (Regensburg, Germany) on behalf of Aldevron Freiburg GmbH (now Genovac, Freiburg, Germany) and were only used to generate VP8*-specific polyclonal antibodies.
Wild animals	The study did not involve wild animals.
Reporting on sex	Only female animals were used for mouse and guinea pig immunogenicity studies.
Field-collected samples	The study did not involve samples collected from the field.
Ethics oversight	Female BALB/cAnNRj mice (Janvier Labs, France) were provided and handled by Preclinics Gesellschaft für präklinische Forschung mbH (Potsdam, Germany). All in-life experimental procedures undertaken during the course of the mouse immunization study were conducted in accordance with German laws and guidelines for animal protection and appropriate local and national approvals as well as in accordance with Directive 2010/63/EU of the European Parliament and of the Council of 22 September 2010 on the protection of animals used for scientific purposes. The mouse experiments were approved by Land Brandenburg, Landesamt für Arbeitsschutz, Vebraucherschutz und Gesundheit (reference number: 2347-14-2018). Mice were acclimated for at least 1 week before any procedures were carried out and were 6-8 weeks old at the start of the study.<br>Female Dunkin Hartley guinea pigs (Envigo RMS, The Netherlands) were provided and handled by Covance Laboratories Ltd. (now Labcorp, Huntingdon, UK). All in-life experimental procedures undertaken during the course of the guinea pig immunization studies were subject to the provisions of the United Kingdom Animals (Scientific Procedures) Act 1986 Amendment Regulations 2012 (the Act). The guinea pig experiments were approved by the local (Huntingdon, UK) Animal Welfare and Ethical Review Body (AWERB) and conducted under the Home Office PPL number 70/8559 (Protocol No. 2). Guinea pigs were acclimated for at least 1 week before any procedures were carried out and were 6-7 weeks old at the start of the first study and 10-11 weeks old at the start of the second study.<br>Female rabbits were provided and handled by Davids Biotechnologie GmbH (Regensburg, Germany) on behalf of Aldevron Freiburg GmbH (now Genovac, Freiburg, Germany). All in-life experimental procedures undertaken during the course of the rabbit immunization study were conducted in accordance with German laws and guidelines for animal protection and appropriate local and national approvals as well as in accordance with Directive 2010/63/EU of the European Parliament and of the Council of 22 September 2010 on the protection of animals used for scientific purposes. The rabbit experiments were approved by the Regional Board Unterfranken, controlled via the district veterinary office Regensburg (reference number: 54-2532.4-04/14). Rabbits were acclimated for at least 1 week before any procedures were carried out and were 10 weeks old at the start of the study.

Note that full information on the approval of the study protocol must also be provided in the manuscript.

Clinical data

Policy information about clinical studies

All manuscripts should comply with the ICMJE guidelines for publication of clinical research and a completed CONSORT checklist must be included with all submissions.

Clinical trial registration	n/a
Study protocol	n/a
Data collection	n/a
Outcomes	n/a

Dual use research of concern

Policy information about dual use research of concern

Hazards

Could the accidental, deliberate or reckless misuse of agents or technologies generated in the work, or the application of information presented in the manuscript, pose a threat to:

No	Yes	
☒	☐	Public health
☒	☐	National security
☒	☐	Crops and/or livestock
☒	☐	Ecosystems
☒	☐	Any other significant area

Experiments of concern

Does the work involve any of these experiments of concern:

No	Yes	
☒	☐	Demonstrate how to render a vaccine ineffective
☒	☐	Confer resistance to therapeutically useful antibiotics or antiviral agents
☒	☐	Enhance the virulence of a pathogen or render a nonpathogen virulent
☒	☐	Increase transmissibility of a pathogen
☒	☐	Alter the host range of a pathogen
☒	☐	Enable evasion of diagnostic/detection modalities
☒	☐	Enable the weaponization of a biological agent or toxin
☒	☐	Any other potentially harmful combination of experiments and agents

ChIP-seq

Data deposition

☐ Confirm that both raw and final processed data have been deposited in a public database such as GEO.

☐ Confirm that you have deposited or provided access to graph files (e.g. BED files) for the called peaks.

Data access links<br>*May remain private before publication.*	n/a
Files in database submission	n/a
Genome browser session<br>(e.g. UCSC)	n/a

Methodology

Replicates	n/a
Sequencing depth	n/a
Antibodies	n/a
Peak calling parameters	n/a
Data quality	n/a
Software	n/a

Flow Cytometry

Plots

Confirm that:

☒ The axis labels state the marker and fluorochrome used (e.g. CD4-FITC).

☒ The axis scales are clearly visible. Include numbers along axes only for bottom left plot of group (a 'group' is an analysis of identical markers).

☒ All plots are contour plots with outliers or pseudocolor plots.

☒ A numerical value for number of cells or percentage (with statistics) is provided.

Methodology

Sample preparation	The induction of antigen-specific T cells was determined using intracellular cytokine staining in combination with flow cytometry. For this purpose, mouse splenocytes were thawed and 2 × 106 cells per well (200 µL) were stimulated for 5-7 hours at 37°C using the following VP8*-specific peptides at 5 µg/mL each: FYIIPRSQE, KYGGRVWTF, VYESTNNSD, FYNSVWTFH, GFMKFYNSV, SDFWTAVIAVEPHVN, TNKTDIWVALLLVEP, and HKRTLTSDTKLAGFM. After 1 h, GolgiPlugTM (BD Biosciences, Cat. 555029) was added in a dilution of 1:200 (50 µL) to the splenocytes to inhibit the secretion of cytokines. After stimulation, splenocytes were centrifuged, resuspended in supplemented medium, and stored overnight at 4°C. The next day splenocytes were washed twice in PBS and stained with LIVE/DEADTM fixable aqua dead cell stain kit (Invitrogen, Cat. L34957) for 30 min at 4°C. After an additional washing step in PBS with 0.5% BSA, cells were surface stained for Thy1.2 (FITC rat anti-mouse CD90.2 (Thy1.2); 1:200; BioLegend, Cat. 140304), CD4 (V450 rat anti-mouse CD4; 1:200; BD Biosciences, Cat. 560468) and CD8 (APC-H7 rat anti-mouse CD8a; 1:100; BD Biosciences, Cat. 560182) and incubated with FcγR-block (rat anti-mouse CD16/CD32; 1:100; Invitrogen, Cat. 14-0161-85) in PBS with 0.5% BSA for 30 min at 4°C. Subsequently, splenocytes were washed and fixed using Cytofix/CytopermTM solution (BD Biosciences, Cat. 554722) for 20 min at RT in the dark. After fixation, cells were washed in perm buffer (PBS, 0.5% BSA, 0.1% Saponin) and stained for IFN-γ (APC rat anti-mouse IFN-γ; 1:100; BD Biosciences, Cat. 554413) and TNF (PE rat anti-mouse TNF alpha, 1:100; Invitrogen, Cat. 12-7321-82) for 30 min at RT. After intracellular cytokine staining, splenocytes were washed in perm buffer and resuspended in PFEA buffer (PBS, 2% FCS, 2 mM EDTA, 0.01% sodium azide).
Instrument	Splenocytes were analyzed by flow cytometry on a Canto II flow cytometer (BD Biosciences).
Software	Flow cytometry data were analyzed using FlowJo software (Tree Star, Inc.; Ashland, OR, USA).
Cell population abundance	No cell sorting was performed for this assay. All samples were analyzed but not sorted into individual populations.
Gating strategy	T cells were characterized as singlets, viable cells, Thy1.2+, CD4+ or CD8+ and subdivided into multifunctional T cell subsets based on their expression of IFN-γ and TNF.

☒ Tick this box to confirm that a figure exemplifying the gating strategy is provided in the Supplementary Information.

Magnetic resonance imaging

Experimental design

Design type	n/a
Design specifications	n/a
Behavioral performance measures	n/a

Acquisition

Imaging type(s)	n/a
Field strength	n/a
Sequence & imaging parameters	n/a
Area of acquisition	n/a

Diffusion MRI ☐ Used ☐ Not used

Preprocessing

Preprocessing software	n/a
Normalization	n/a
Normalization template	n/a
Noise and artifact removal	n/a
Volume censoring	n/a

Statistical modeling & inference

Model type and settings	n/a
Effect(s) tested	n/a

Specify type of analysis: ☐ Whole brain ☐ ROI-based ☐ Both

Statistic type for inference (See Eklund et al. 2016)	n/a
Correction	n/a

Models & analysis

n/a	Involved in the study
☐	☐ Functional and/or effective connectivity
☐	☐ Graph analysis
☐	☐ Multivariate modeling or predictive analysis

Functional and/or effective connectivity	n/a
Graph analysis	n/a
Multivariate modeling and predictive analysis	n/a
